# Exercise-induced hippocampal anti-inflammatory response in aged rats

**DOI:** 10.1186/1742-2094-10-61

**Published:** 2013-05-10

**Authors:** Sérgio Gomes da Silva, Priscila Santos Rodrigues Simões, Renato Arruda Mortara, Fulvio Alexandre Scorza, Esper Abrão Cavalheiro, Maria da Graça Naffah-Mazzacoratti, Ricardo Mario Arida

**Affiliations:** 1 Departamento de Fisiologia, Universidade Federal de São Paulo, Rua Botucatu 862, Ed. Ciências Biomédicas, 5 andar. Vila Clementino, São Paulo, SP, Zip Code 04023-900, Brazil; 2 Faculdade do Clube Náutico Mogiano, Zip Code 08773-000,, Mogi das Cruzes, Brazil; 3 Department of Neurology and Neurosurgery, Universidade Federal de São Paulo, São Paulo, Zip Code 04023-900, Brazil; 4 Department of Microbiology, Immunology and Parasitology, Universidade Federal de São Paulo, São Paulo, Zip Code 04023-900, Brazil

**Keywords:** Exercise, Treadmill, Brain, Aging, Plasticity, Cytokine, Inflammation

## Abstract

Aging is often accompanied by cognitive decline, memory impairment and an increased susceptibility to neurodegenerative disorders. Most of these age-related alterations have been associated with deleterious processes such as changes in the expression of inflammatory cytokines. Indeed, higher levels of pro-inflammatory cytokines and lower levels of anti-inflammatory cytokines are found in the aged brain. This perturbation in pro- and anti-inflammatory balance can represent one of the mechanisms that contribute to age-associated neuronal dysfunction and brain vulnerability. We conducted an experimental study to investigate whether an aerobic exercise program could promote changes in inflammatory response in the brains of aged rats. To do so, we evaluated the levels of tumor necrosis factor alpha (TNFα), interleukin 1 beta (IL1β), interleukin 6 (IL6) and interleukin 10 (IL10) in the hippocampal formation of 18 month old rats that underwent treadmill training over 10 consecutive days. Quantitative immunoassay analyses showed that the physical exercise increased anti-inflammatory cytokine levels IL10 in the hippocampal formation of aged rats, when compared to the control group. The hippocampal levels of pro-inflammatory cytokines IL1β, IL6 and TNFα were not statistically different between the groups. However, a significant reduction in IL1β/IL10, IL6/IL10 and TNFα/IL10 ratio was observed in the exercised group in relation to the control group. These findings indicate a favorable effect of physical exercise in the balance between hippocampal pro- and anti-inflammatory during aging, as well as reinforce the potential therapeutic of exercise in reducing the risk of neuroinflammation-linked disorders.

## Introduction

Aging is often accompanied by cognitive decline, memory impairment and an increased susceptibility to neurodegenerative disorders [1]. Most of these age-related alterations have been associated with deleterious processes such as changes in cytokine expression [2]. Cytokines are cell-signaling proteins secreted to mediate the response of the body’s defense system to injury, and to regulate diverse inflammatory processes. These cell-signaling proteins include pro-inflammatory cytokines, such as TNFα, IL1β and IL6, and anti-inflammatory cytokines, such as IL10. Short-term inflammatory reactions in response to injuries are adaptive and essential for survival, but chronic inflammation can be harmful. In the aging brain, pro-inflammatory cytokines have been found to be chronically increased [3]. Higher IL6 levels were observed in the cortex, hippocampal formation and cerebellum of aged mice when compared to juvenile and adult mice [4]. Additionally, reduced IL10 levels have been detected in the aged brain [5]. Evidence indicates that age-related decline in IL10 levels may contribute to the increased expression of brain IL6 in aged mice [5]. This perturbation in pro- and anti-inflammatory balance may represent one of the mechanisms that contribute to age-related neuronal dysfunction and the brain’s vulnerability to diseases [6]. Indeed, increased levels of pro-inflammatory cytokines are reported in neurodegenerative disorders such as Parkinson’s disease, Alzheimer’s disease and other chronic conditions [7]. Consequently, pharmacological and non-pharmacological interventions targeting the cytokines and their signaling pathways have been suggested for therapeutic purposes [8].

There is a great deal of evidence showing the capacity of physical exercise to reduce (or ameliorate) the risk of common age-associated disorders [9,10]. Exercise may be a potential intervention to improve the cognitive performance of the elderly [11], and to reduce the onset or progression of neurodegenerative disorders such as Parkinson’s disease [12] and Alzheimer’s disease [13]. These beneficial effects of exercise during aging should be related to changes in cytokine brain expression. Recent investigation showed that exercise can shift the inflammatory response in the brain of Tg2576 Alzheimer mice [14]. In view of these observations, we conducted a study to investigate the levels of TNFα, IL1β, IL6 and IL10 in the hippocampal formation - a highly plastic region of the brain that is linked to cognitive and emotional processes - of senescent rats submitted to an aerobic exercise program.

## Methods

### Exercise paradigm

Eighteen month-old male Wistar rats (n = 20) were used in this study. The colony room was maintained at 21 ± 2°C with a 12 h light/dark schedule (light: 7 am until 7 pm), and food and water were provided *ad libitum* throughout the experimental period. The rats were divided into two groups, exercise and control (n = 10 rats in each group). Animals in the exercise group were familiarized with the apparatus for three days by placing them on a treadmill (Columbus instruments) for 5 minutes/day at a speed of 8 m/minute at a 0% degree incline. Electric shocks were used sparingly to motivate the rats to run. To provide a measure of trainability, we rated each animal’s treadmill performance on scale of 1 to 5 [1, refused to run; 2, below average runner (sporadic, stop and go, wrong direction); 3, average runner; 4, above average runner (consistent runner occasionally fell back on the treadmill); 5, good runner (consistently stayed at the front of the treadmill)]. Animals with a mean rating of 3 or higher were included in the exercise group. If any animal was excluded from the exercise group it would not form the control group. This procedure was used to exclude possible differences in stress levels between animals [15]. However, no animal in this study had to be excluded from the exercise group (all animals submitted to physical training were good runners). Afterwards, animals were submitted to an aerobic exercise program of 10 days on the treadmill. Each training session started with a 5-minute warm-up at 8 m/minute. Running time and speed were gradually increased from 12 m/minute at 10 minutes during the first sessions to 15 m/minute at 30 minutes in the following training days. The training period occurred between 9 and 10 am. Animals in the control group were transferred to the experimental room and handled in the same way as animals in the exercise group (privation of water and food during treadmill exercise). All experimental protocols were approved by the ethics committee of the Universidade Federal de São Paulo (#0607/09) and all efforts were made to minimize animal suffering in accordance with the proposals of International Ethical Guideline for Biomedical Research (CIOMS, 1985).

### Immunofluorescence

Immunofluorescence was performed to verify the hippocampal cytokine spatial distribution. Aged animals from both the exercise and control groups (n = 4 from each group) were deeply anesthetized (Tionembutal, 50 mg-kg, intraperitoneally) and perfused transcardially with solution of 0.01 M PBS, followed by a solution containing 4% formaldehyde in 0.1 M phosphate buffer (PB), pH 7.4. Animals from the exercise group were killed 1 h after the last exercise session. Animals from control group were killed after the same period of time as in exercised animals. After perfusion, the brains were removed immediately from the skull and post-fixed in 4% paraformaldehyde in PB for 24 h. The brains were then cut coronally with a vibratome (Leica) in 50 μm-thick slices and stored at −20°C in the biological tissue bank in our laboratory (for preservation of tissue). To inhibit the formation of ice crystals that damage the structure of cells, the slices were maintained in an antifreeze solution containing 30% sucrose, 1% polyvinylpyrrolidone 40 (PVP-40) and 30% ethylene glycol in PB (pH 7.2). Afterwards, hippocampal slices (bregma −2.8/−3.3 mm; [16]) were rinsed in PBS and blocked and permeabilized for 20 minutes in PBS solution containing 0.01% saponin and 1% bovine albumin. The slices were then incubated for 48 h with the respective primary antibodies (IL1β (1:100; IBL), IL6 (1:100; IBL), IL10 (1:100; R&D) and TNFα (1:100; IBL)) previously diluted in the blocking solution. For each primary antibody, we used two slices from each animal from both groups (exercise and control). Subsequently, all slices were rinsed in PBS containing 0.01% saponin and 1% albumin and incubated for 30 minutes with secondary antibodies (1:200) conjugated to AlexaFluor® 488 (IL1β, IL6 and TNFα; green) or 564 (IL10; red) diluted in PBS containing 1 μm 4′,6-diamidino-2-phenylindole (DAPI; Sigma Chemical Co.). Finally, the slices were washed in PBS, mounted on slides with Vectashield (Merck). Regions of Ammon’s horn and dentate gyrus of the hippocampal formation from studied groups were then imaged in a confocal laser scanning system from BioRad 1024UV attached to a Zeiss Axiovert 100 microscope using a 40× 1.2 NA PlanApochromatic water immersion lens.

### Immunoassay

The immunoassay was performed to determine hippocampal cytokine levels. The hippocampal formation of aged animals from the exercise and control groups (n = 6 from each group) was removed immediately after decapitation and homogenized in 0.01 M Tris hydrochloride (pH 7.6) containing 5.8% sodium chloride, 10% glycerol, 1% Nonidet P40 (NP-40), 0.4% of ethylenediamine tetraacetic acid (EDTA) and protease inhibitors. Animals from the exercise group were killed 1 h after the last exercise session. Animals from the control group were killed after the same time period as exercised animals. Samples were sonicated and stored at −80°C. Subsequently, samples were centrifuged for 5 minutes at 10,000 (×*g*) at 4°C and concentrations determined with a Millipore multiplex Rat Cytokine Kit (IL1β, IL6, TNFα, and IL10) on the Luminex® xMAP® platform. Longitudinal controls were used to assess the interassay variation. Statistical analyses between control and exercise groups were conducted by Student’s *t*-test. The IL1β/IL10, IL6/IL10 and TNFα/IL10 ratios were calculated to verify the relationship between the pro- and anti-inflammatory cytokines. All values were considered significant when *P* was <0.05. Data are presented as mean and standard error of the mean (± SEM).

## Results

**Figure 1:**
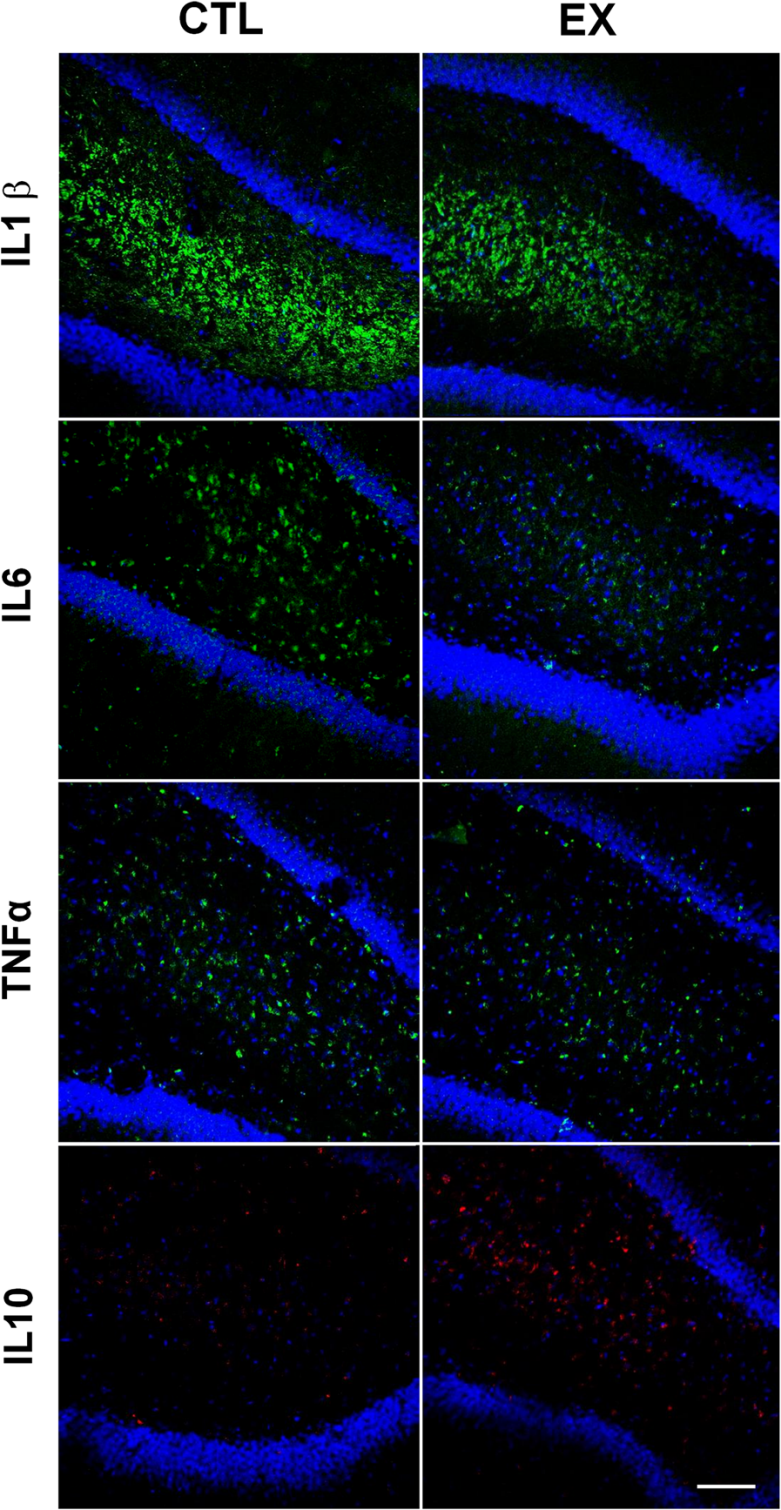
**Pro- (IL1β, IL6 and TNFα; green) and anti-inflammatory (IL10; red) immunostaining in the dentate gyrus (marked with DAPI; blue) of aged rats from control (CTL) and exercise (EX) groups.** Scale bar = 100 μm. DAPI, 4′,6-diamidino-2-phenylindole.

**Figure 2:**
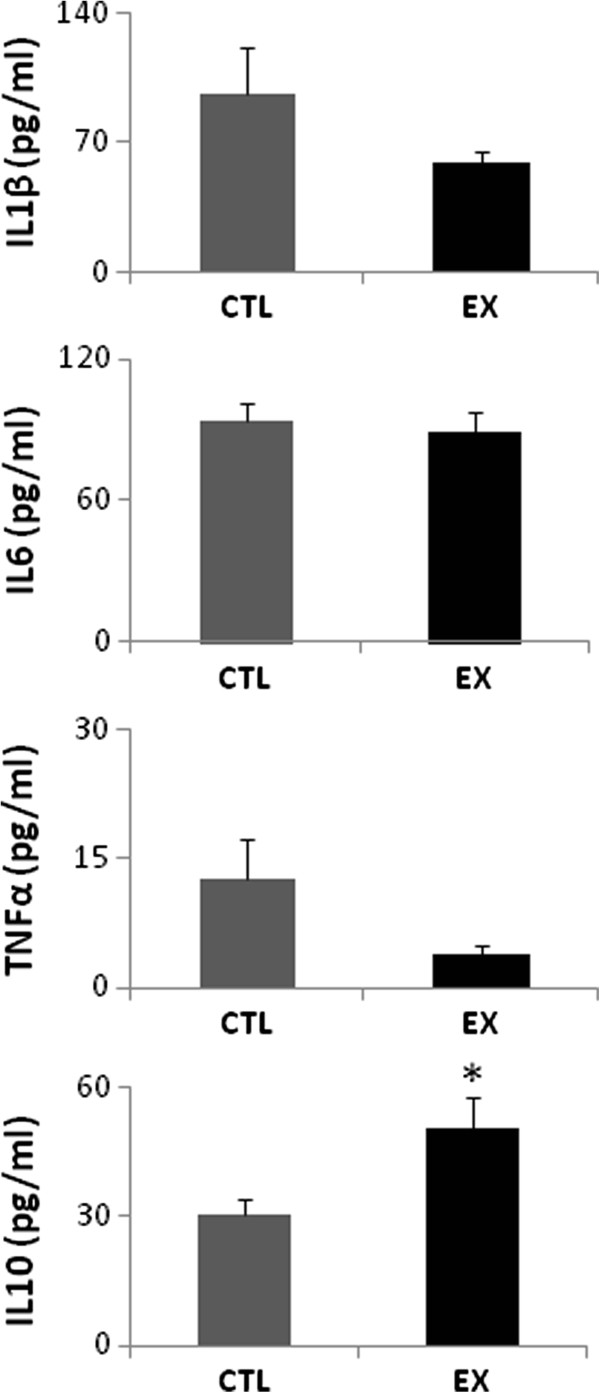
**IL1β, IL6, TNFα and IL10 levels in the hippocampal formation of aged rats from control (CTL) and exercise (EX) groups.** A significant increase in the hippocampal IL10 levels was found in the EX group when compared to the CTL group (*P <0.05; Student’s *t*-test).

**Figure 3:**
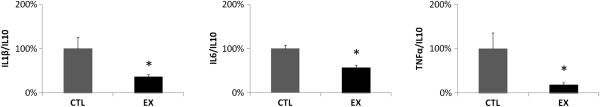
**IL1β/IL10, IL6/IL10 and TNFα/IL10 ratios in the hippocampal formation of aged rats from control (CTL) and exercise (EX) groups.** The data are presented in percentage (CTL group = 100%). *Significant different from CTL group (*P* <0.05; Student’s *t*-test).

The hippocampal cytokine spatial distribution in this study was similar to previously described patterns [17]. In aged rats from both groups, the pro- (IL1β, IL6 and TNFα) and anti-inflammatory (IL10) immunostaining was mostly located within or in the vicinity of the pyramidal cell layer of Ammon’s horn and in polymorphic layers of the dentate gyrus (Figure 1). An apparent change in hippocampal inflammatory immunostaining was observed between the exercise and control groups when qualitatively analyzed by immunofluorescence. When quantitatively analyzed by immunoassay, a significant increase in the IL10 levels was detected in the hippocampal formation of aged rats from the exercise group (50.33 ± 7.05 pg/ml, *P* <0.05) compared with those of the control group (30.34 ± 3.81 pg/ml) (Figure 2). No significant differences in hippocampal IL1β, IL6 and TNFα levels were found between the two groups (exercise group: IL1β = 58.47 ± 7.14 pg/ml, IL6 = 88.64 ± 9.25 pg/ml and TNFα = 3.88 ± 0.96 pg/ml; control group: IL1β = 95.62 ± 25.69 pg/ml, IL6 = 93.62 ± 7.60 pg/ml and TNFα = 12.61 ± 4.54 pg/ml; *P* >0.05) (Figure 2). However, a significant reduction in IL1β/IL10, IL6/IL10 and TNFα/IL10 ratio was observed in the exercise group (IL1β/IL10 = 1.2 ± 0.12 pg/ml, IL6/IL10 = 1.76 ± 0.18 pg/ml and TNFα/IL10 = 0.08 ± 0.01 pg/ml; *P* <0.05) when compared to the control group (IL1β/IL10 = 3.2 ± 0.84 pg/ml, IL6/IL10 = 3.09 ± 0.25 pg/ml and TNFα/IL10 = 0.42 ± 0.15 pg/ml) (Figure 3).

## Discussion

The present study investigated the hippocampal levels of TNFα, IL1β, IL6 and IL10 in senescent rats submitted to an aerobic exercise program of 10 days on a treadmill. Quantitative immunoassay analyses showed that physical exercise increased the IL10 levels in the hippocampal formation of aged rats when compared to control rats. The hippocampal levels of pro-inflammatory cytokines IL1β, IL6 and TNFα were not statistically different between studied groups. Studies using young rodents have shown that exercise can alter brain expression of pro-inflammatory cytokines [18,19]. It was reported that progressive exercise training led to reductions in IL1β concentrations in the hippocampus and IL6 concentrations in the cerebellum [18]. On the other hand, other studies have not found significant differences in IL1β, IL6 and TNFα expression in the young brain of sedentary and trained rodents [20,21]. The latter results accord with our data and with previous findings in non-transgenic aged mice with free access to running wheels for 3 weeks [14]. However, it is important to point out that a significant reduction in the hippocampal IL1β/IL10, IL6/IL10 and TNFα/IL10 ratio was detected in aged animals from the exercise group as compared with the control group. Taken together, these findings indicate a favorable effect of physical exercise on the hippocampal pro- and anti-inflammatory balance of aged rats.

Inflammatory cytokines can exert negative or positive effects on brain functions. These effects are dependent on numerous factors, including the type of cytokine produced, the functional state and type of stimulated cells, and the concentration and duration of exposure to the cytokines [6]. In particular, the aged brain has been characterized by increased levels of pro-inflammatory cytokines [3]. The upregulation of pro-inflammatory cytokines may contribute to unsuccessful maintenance of neuronal communication and to cell death by modulating the NMDA and AMPA receptors [6]. The overactivation of these glutamate receptors can induce neurodegenerative processes and amplify neuronal vulnerability through increased intracellular calcium concentration, strongly deregulated in aging [22]. Furthermore, it has been described that pro-inflammatory cytokines impair the aged brain’s ability to maintain hippocampal long-term potentiation (LTP) [23], a critical physiological process involved in memory consolidation. The inflammation-induced LTP impairment is accompanied by enhanced activity of stress-activated protein kinases and reactive oxygen species [23]. Interestingly, promising data have shown that inflammation-linked LTP impairment can be reversed by intracerebroventricular infusion of the anti-inflammatory cytokine IL10 [24].

As mentioned before, anti-inflammatory cytokines are significantly reduced in the aged brain [5]. This anti-inflammatory downregulation has been related to increased expression of pro-inflammatory cytokines and neuronal injury [2,5]. Indeed, IL10 knockout mice are more sensitive to neuronal damage and behavioral deficits [25,26]. These findings suggest that IL10 reduction in the aged brain might result in neuronal dysfunction and vulnerability. Thus, interventions to increase IL10 levels could have great therapeutic value. In the present study, we observed that an aerobic exercise program of 10 days enhanced IL10 levels and reduced the pro-/anti-inflammatory cytokine ratio in the hippocampal formation of aged rats. The importance of anti-inflammatory response induced by exercise indicates its potential therapeutic action for age-related brain inflammatory imbalance as well as to reduce the risk of neuroinflammation-linked disorders.

Some factors could contribute to this anti-inflammatory effect of exercise in the aging brain. These factors may include a large number of cell-signaling proteins induced by exercise, which are known to be associated with neuronal survival and proliferation, and synaptic plasticity [27]. Among them, growth-related proteins such as neurotrophins have been considered the most likely candidates in mediating the effects of exercise on brain functions [28]. Neurotrophins are a heterogeneous group of endogenous proteins secreted to regulate cellular processes of proliferation, development and differentiation. These proteins are synthesized by both neurons and glial cells and allow neurons to receive adequate nutrition to grow, develop or regenerate themselves. Previous data in young rats show that a few days of exercise result in a significant upregulation of neurotrophins such as nerve growth factor (NGF) and brain-derived neurotrophic factor (BDNF) [29]. Considering recent findings showing that stimulation of dendritic cells with NGF and BDNF induces IL10 release [30], it is possible that our data could also be correlated to the increase of NGF and BDNF levels in the hippocampal formation of aged rats submitted to physical exercise. In support of this idea, it has been observed that short bouts of exercise may increase the hippocampal levels of the BDNF gene and protein in aged rats [31]. Nevertheless, further studies are needed to verify if the increase of exercise-induced anti-inflammatory response during aging is linked to neurotrophin levels and whether the IL10 levels remain increased after discontinuation of the physical training.

## Abbreviations

BDNF: brain-derived neurotrophic factor; DAPI: 4′,6-diamidino-2-phenylindole; EDTA: ethylenediamine tetraacetic acid; IL: interleukin; LTP: long-term potentiation; NGF: nerve growth factor; PB: phosphate-buffered solution; PBS: phosphate-buffered saline; PVP: polyvinylpyrrolidone; SEM: standard error of the mean; TNFα: tumor necrosis factor alpha.

## Competing interests

The authors declare that they have no competing interests.

## Authors’ contributions

SGS and RMA designed the experiments, performed analysis of data and wrote the manuscript. PSRS and MGNM provided expertise on inflammation, and contributed to immunofluorescence analysis and discussions. RAM acquired and processed images, and contributed to discussion. FAS and EAC participated in the manuscript preparation and discussions. All authors read and approved the final manuscript.
